# Supplementary material for: Motor unit discharge properties are modestly influenced by menstrual cycle-related fluctuations in sex hormone concentrations
Source: bioRxiv. 2026 Jan 20:2026.01.16.699975. Preprint. [Version 1] doi: 10.64898/2026.01.16.699975 (PMC12871738; doi:10.64898/2026.01.16.699975)
Supplement: 1 [file NIHPP2026.01.16.699975V1-supplement-1.pdf]

bioRxiv preprint doi: https://doi.org/10.64898/2026.01.16.699975; this version posted January 20, 2026. The copyright holder for this preprint (which was not certified by peer review) is the author/funder, who has granted bioRxiv a license to display the preprint in perpetuity. It is made available under aCC-BY 4.0 International license.

	Site 1	Site 2	Site 3
**Ethical Approval**	Northumbria University ethics committee (2025-2878-11132).	Northwestern University Institutional Review Board (STU00216360).	Nottingham Trent University invasive ethics approval (1606166).
**Experimental Apparatus**	Adjustable chair; ankle positioned at 95°; ankle strapped to TF 022 transducer (CCt Transducers, Italy) housed in a wooden ankle dynamometer (OT Bioelettronica, Italy); force signals amplified and sampled at 2048 Hz.	Biodex chair with footplate attachment; ankle at 95-100°, hip at 100°, knee fully extended; Systems 2 Dynamometer (Biodex Medical Systems, USA); torque sampled at 2048 Hz and low-pass filtered offline.	Custom-built chair with hip at 110°, knee fully extended; ankle at 95-100°, securely strapped to force dynamometer (purpose-built strain gauge, RS125 Components Ltd, Corby UK) with non-compliant straps around the mid foot; force signals sampled at 2000Hz.
**HDsEMG Preparation**	Skin shaved, abraded with Nuprep gel, and cleaned with 70% isopropyl alcohol; reference electrode strap placed around the right wrist; ground electrode strap placed around right ankle	Skin shaved and lightly abraded with EVERI paste; reference electrode strap placed around the right ankle; ground electrode placed on the patella	Skin prepared using razor for initial shaving and then lightly abrased with paper and (3M red dot trace prep) Nuprep gel; ground electrode placed around the right ankle. (no reference electrode)
**HDsEMG Acquisition**	Sampled at 2048 Hz, amplified 150x, band-pass filtered at 10-500 Hz using Quattrocento amplifier (OT Bioelettronica, Italy); HDsEMG recorded in monopolar mode; EMG and torque signals collected and synchronized through Quattrocento.	Sampled at 2048 Hz, amplified 150x, band-pass filtered at 10-500 Hz using Quattrocento amplifier (OT Bioelettronica, Italy); HDsEMG recorded in monopolar mode; EMG and torque signals synced using TTL pulse at onset of each trial.	10 subjects' data acquired with Sessantaquattro (OT Bioelettronica, Italy): HDsEMG recorded in monopolar mode sampled at 2000Hz, amplified 256x, band pass filtered at 10-500Hz using the 16-bit wireless amplifier. EMG and torque signals collected and synchronized through Sessantaquattro.<br><br>10 subjects' data acquired with Sessantaquattro+ (OT Bioelettronica, Italy): HDsEMG recorded in monopolar mode sampled at 2000Hz, amplified 256×, band pass filtered at 10-500Hz using the 16-bit wireless amplifier. EMG and torque signals collected and synchronized through Sessantaquattro+; Lag time between EMG and torque signals were estimated using OTBiolab V25.5 offline and where present, the force signal was corrected to realign with EMG.

bioRxiv preprint doi: https://doi.org/10.64898/2026.01.16.699975; this version posted January 20, 2026. The copyright holder for this preprint (which was not certified by peer review) is the author/funder, who has granted bioRxiv a license to display the preprint in perpetuity. It is made available under aCC-BY 4.0 International license.

**Experimental Protocol**	Standardized warm-up at 25%, 50%, and 75% of perceived maximum effort; three MVCs performed with the highest value used to set contraction intensity; additional MVC if final >5% of previous values.	Three MVCs performed; average value used for analysis; if the final trial produced the highest torque, additional trials were performed as needed.	Standardized warm-up at 25%, 50% and 70% of perceived maximum effort; three MVCs performed with highest value used to set contraction intensity; additional MVC conducted if final MVC exceeded previous by >10%.
**Motor Unit Decomposition**	Offline analyses of the multichannel surface EMG signal were performed within MATLAB (R2022b, The Mathworks Inc., MA, USA). Monopolar signals were digitally band pass filtered with a Butterworth filter (20-500 Hz) and channels excluded if they exhibited a low pulse-to-noise ratio using the MATLAB tool DEMUSE (version 6.1, University of Maribor, Slovenia). The well-validated Convolution Kernal Compensation algorithm based on blind source separation (Holobar & Zazula, 2007; Holobar & Farina, 2021) was used to calculate a motor unit filter, giving an estimate of motor unit spike trains. The filter was manually edited and reapplied to optimize the motor unit spike train estimation and after this only motor unit spike trains with a reliable discharge pattern and a pulse-to-noise ratio ≥ 28 dB were kept for analysis.	After data collection, HDsEMG signals were manually inspected; channels with substantial artifacts or saturation were removed; individual motor unit spike trains were decomposed using a convolutive blind-source separation algorithm with a silhouette threshold of 0.87 (Negro *et al.*, 2016)	After data collection, HDsEMG signals were digitally band pass filtered using a Butterworth filter (20-500Hz) and channels excluded that exhibited a low pulse-to-noise ratio using the MATLAB tool DEMUSE (version 6.1, University of Maribor, Slovenia). The convolution Kernel compensation algorithm based on blind source separation (Holobar & Zazula, 2007; Holobar & Farina, 2021) was used to calculate motor unit filters and estimate corresponding spike trains. Only motor unit spike trains with a pulse-to-noise ratio ≥ 28 dB were included for further analysis.
**Blood Draw**	Samples collected post-session using 10 ml serum vacutainers with silica particles; coagulated at room temperature for 2 hours; serum aliquoted and stored at −80 °C.	Blood samples collected pre-session by trained phlebotomists at the outpatient laboratory at Northwestern Medicine; processed using standard clinical protocols.	Blood samples collected pre-session (fasted sample) by a trained phlebotomist; two 10 ml serum vacutainers with silica particles (20ml total); collected from antecubital vein; rested and coagulated for 25 mins then centrifuged at 2000 × g for 15 min at 4 °C then serum aliquoted into 1 ml tubes and stored at −80 °C until analysis.

bioRxiv preprint doi: https://doi.org/10.64898/2026.01.16.699975; this version posted January 20, 2026. The copyright holder for this preprint (which was not certified by peer review) is the author/funder, who has granted bioRxiv a license to display the preprint in perpetuity. It is made available under aCC-BY 4.0 International license.

**Hormone analysis**	Serum estradiol and progesterone were measured using electro-chemiluminescence immunoassay (ECLIA) on the COBAS e601 automated platform (Roche Diagnostics, Mannheim, Germany). The inter-assay coefficient of variations (CV) were ≤3% within their respective analytical working ranges.	Serum estradiol and progesterone concentrations were measured by an outpatient clinical laboratory using a chemiluminescent immunoassay on the Beckman Coulter DxI System (Beckman Coulter, Brea, CA, USA), following the laboratory's standard procedures.	Serum estradiol and progesterone were measured using electro-chemiluminescence immunoassay (ECLIA) on the COBAS e601 automated platform (Roche Diagnostics, Mannheim, Germany). The inter-assay coefficient of variations (CV) were ≤3% within their respective analytical working ranges.

Table A1. Experimental setup, HDsEMG procedures, motor unit decomposition methods, and hormone analysis across each testing site.
